# ^11^C-*Para*-aminobenzoic acid PET imaging of *S*. *aureus* and MRSA infection in preclinical models and humans

**DOI:** 10.1172/jci.insight.154117

**Published:** 2022-01-11

**Authors:** Alvaro A. Ordonez, Matthew F.L. Parker, Robert J. Miller, Donika Plyku, Camilo A. Ruiz-Bedoya, Elizabeth W. Tucker, Justin M. Luu, Dustin A. Dikeman, Wojciech G. Lesniak, Daniel P. Holt, Robert F. Dannals, Lloyd S. Miller, Steven P. Rowe, David M. Wilson, Sanjay K. Jain

**Affiliations:** 1 Center for Infection and Inflammation Imaging Research and; 2 Department of Pediatrics, Johns Hopkins University School of Medicine, Baltimore, Maryland, USA.; 3 Department of Radiology and Biomedical Imaging, UCSF, San Francisco, California, USA.; 4 Department of Dermatology,; 5 Russell H. Morgan Department of Radiology and Radiological Sciences, and; 6 Department of Anesthesiology and Critical Care Medicine, Johns Hopkins University School of Medicine, Baltimore, Maryland, USA.

**Keywords:** Infectious disease, Bacterial infections, Diagnostic imaging

## Abstract

Tools for noninvasive detection of bacterial pathogens are needed but are not currently available for clinical use. We have previously shown that *para*-aminobenzoic acid (PABA) rapidly accumulates in a wide range of pathogenic bacteria, motivating the development of related PET radiotracers. In this study, ^11^C-PABA PET imaging was used to accurately detect and monitor infections due to pyogenic bacteria in multiple clinically relevant animal models. ^11^C-PABA PET imaging selectively detected infections in muscle, intervertebral discs, and methicillin-resistant *Staphylococcus aureus*–infected orthopedic implants. In what we believe to be first-in-human studies in healthy participants, ^11^C-PABA was safe, well-tolerated, and had a favorable biodistribution, with low background activity in the lungs, muscles, and brain. ^11^C-PABA has the potential for clinical translation to detect and localize a broad range of bacteria.

## Introduction

Bacterial infections, which cause serious, often life-threatening infections, remain a major threat to human health. The costs of treating hospital-acquired infections and infections due to multidrug resistant organisms (MDROs), which are increasing in prevalence, are staggering and a major burden to health care (1). Therefore, rapid and accurate diagnosis of bacterial infections is essential for early interventions and the rational use of antibiotics.

To address this clinical challenge, we hypothesized that metabolism-targeted small molecules that are selectively incorporated into bacteria could be radiolabeled and detected using PET (1, 2). These bacteria-specific imaging agents would provide noninvasive measurements of molecular pathways and could be used in combination with structural imaging (CT or MRI) to provide an anatomic reference to detect the site and extent of the infection.

We have previously demonstrated that *para*-aminobenzoic acid (PABA), selectively metabolized via the bacterial folate pathway, rapidly accumulates in a wide range of pathogenic bacteria and their respective clinical strains, including MDROs (3–5). PABA is nontoxic and has been used extensively in humans as a marker to distinguish between complete and incomplete 24-hour urine collections (6). In the current study, we evaluated ^11^C-PABA PET imaging as a noninvasive tool to detect bacterial infections in rabbit and rat models, including methicillin-resistant *Staphylococcus aureus* (MRSA) prosthetic joint infection. We also performed first-in-human studies to evaluate the biodistribution and radiation dosimetry of ^11^C-PABA PET imaging in healthy human volunteers.

## Results

### ^11^C-PABA PET imaging can selectively localize sites of infection.

**Figure 1:**
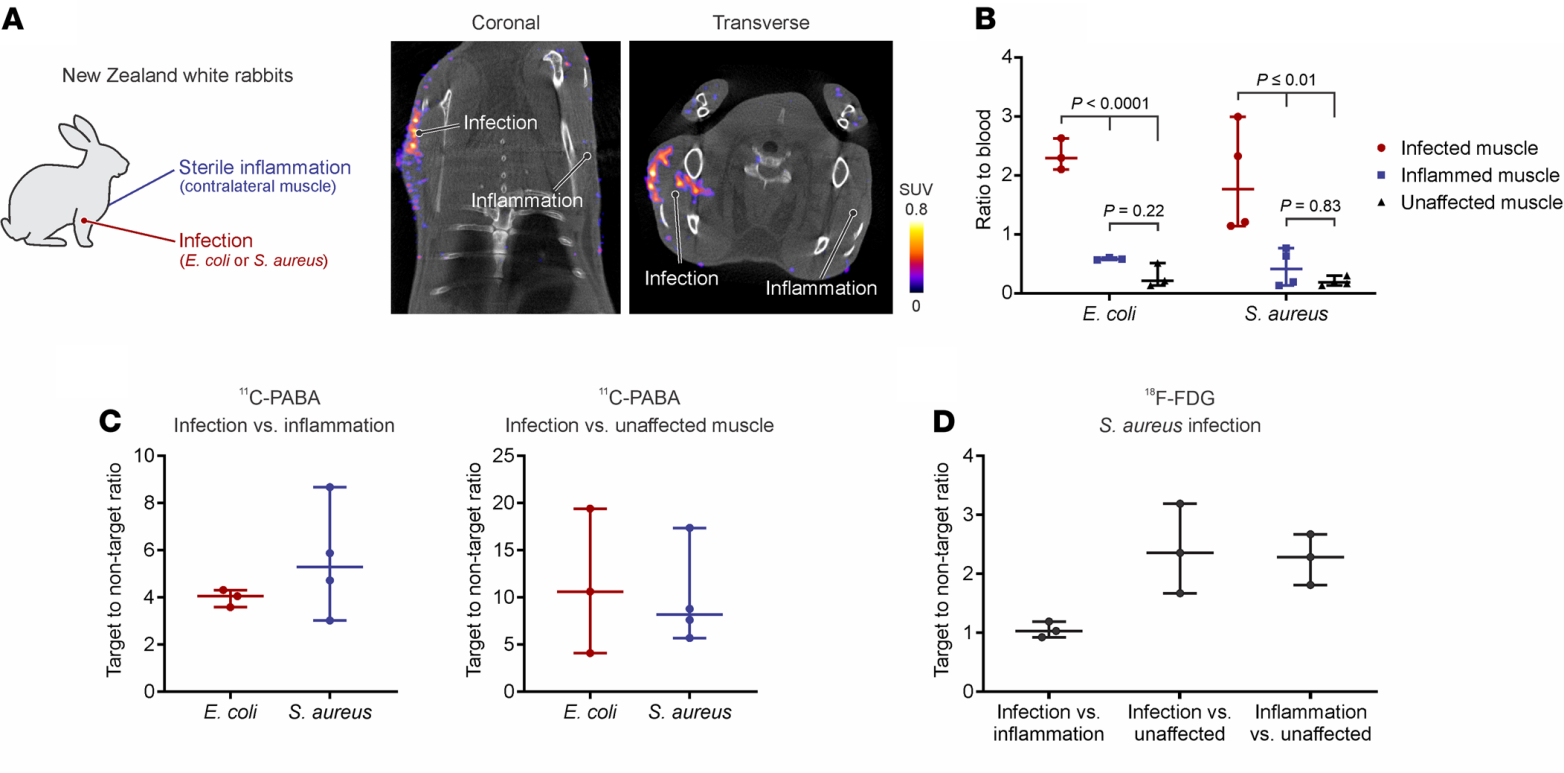
^11^C-PABA PET/CT imaging in a rabbit myositis model. (**A**) New Zealand white rabbits were infected with live bacteria (*E*. *coli* or *S*. *aureus*) in the right triceps and injected with heat-killed bacteria at 10 times the level of live bacteria in the contralateral triceps (left). Coronal and traverse ^11^C-PABA PET/CT imaging of a representative *E*. *coli*–infected rabbit (right). The ^11^C-PABA PET signal was observed at the site of infection (live bacteria) with minimum signal associated with the site of sterile inflammation (heat-killed bacteria). (**B**) The ^11^C-PABA PET signal in rabbits infected with *E*. *coli* (*n* = 3) and *S*. *aureus* (*n* = 4), quantified as ratio to blood. (**C**) Comparison of the volume of interest (VOI) quantification of ^11^C-PABA PET, determined as the infection vs. inflammation target-to-nontarget (TNT) ratio of *E*. *coli* and *S*. *aureus*–infected animals (left). Comparison of the ^11^C-PABA PET infection vs. unaffected muscle TNT ratio of *E*. *coli* and *S*. *aureus*–infected animals (right). (**D**) TNT ratio of *S*. *aureus*–infected animals imaged with ^18^F-FDG PET (*n* = 3). All Data are represented as the median ± IQR. Statistical comparisons were performed using a 1-way ANOVA with Tukey’s multiple-comparisons test.

To evaluate if ^11^C-PABA was able to accurately determine the infection site and differentiate it from sterile inflammation, we used New Zealand white rabbits with experimentally induced myositis due to *E*. *coli* or *Staphylococcus aureus* (Figure 1A). Live bacteria (*E. coli* or *S. aureus*) were injected into the right triceps of rabbits, and 10 times more heat-inactivated bacteria were injected on the contralateral extremity, which was used as control (sterile inflammation). Three days after infection, ^11^C-PABA PET imaging could localize sites of bacterial infections and differentiate them from sterile inflammation (Figure 1A). The bacterial burden after completion of imaging at the infection sites was 8.15 ± 1.4 and 9.24 ± 0.47 log_10_ CFU for the *E*. *coli* and *S*. *aureus* rabbit cohorts, respectively. To quantify the PET signal, spherical volumes of interest (VOIs) were drawn at the site of infection, the left ventricle of the heart (as a surrogate for blood), and the contralateral extremity (sterile inflammation control) to measure the ratio to blood and the target-to-nontarget tissue (TNT) ratio, respectively. The ^11^C-PABA PET signal in the *E*. *coli* and *S*. *aureus* models, quantified as ratio to blood, was significantly higher at the infection site compared with sterile inflammation (*P ≤* 0.01) (Figure 1B). Conversely, there were no differences in ^11^C-PABA PET signal between the site of sterile inflammation and unaffected muscle. The TNT ratio for ^11^C-PABA PET in the *E*. *coli*–infected muscle was 4.05 (IQR, 3.59–4.32) (Figure 1C). In the rabbits infected with *S*. *aureus*, the TNT ratio for ^11^C-PABA PET was 5.30 (IQR, 3.02–8.67) (Figure 1C). When compared with an unaffected muscle (paravertebral muscle) from the same animal, the TNT ratio for ^11^C-PABA PET at the infected muscle sites was 10.61 (IQR, 4.09–19.40) and 8.20 (IQR, 7.62–17.36) for *E*. *coli* and *S*. *aureus,* respectively (Figure 1C). As a comparison, the same animals infected with *S*. *aureus* were imaged with 2-deoxy-2-[^18^F]fluoro-ᴅ-glucose (^18^F-FDG) PET/CT. The ^18^F-FDG PET signal was similar between the site of infection and the contralateral sterile inflammation, with a TNT of 1.03 (IQR, 0.92–1.19) (Figure 1D). Similarly, the animals infected with *E*. *coli* were imaged with 2-deoxy-2-[^18^F]fluoro-ᴅ-sorbitol (^18^F-FDS) PET/CT (7), with an infection compared with sterile inflammation TNT ratio of 3.16 (IQR, 3.14–3.49) (Supplemental Figure 1; supplemental material available online with this article; https://doi.org/10.1172/jci.insight.154117DS1).

### ^11^C-PABA PET imaging can detect MRSA infections in a prosthetic joint model.

**Figure 2:**
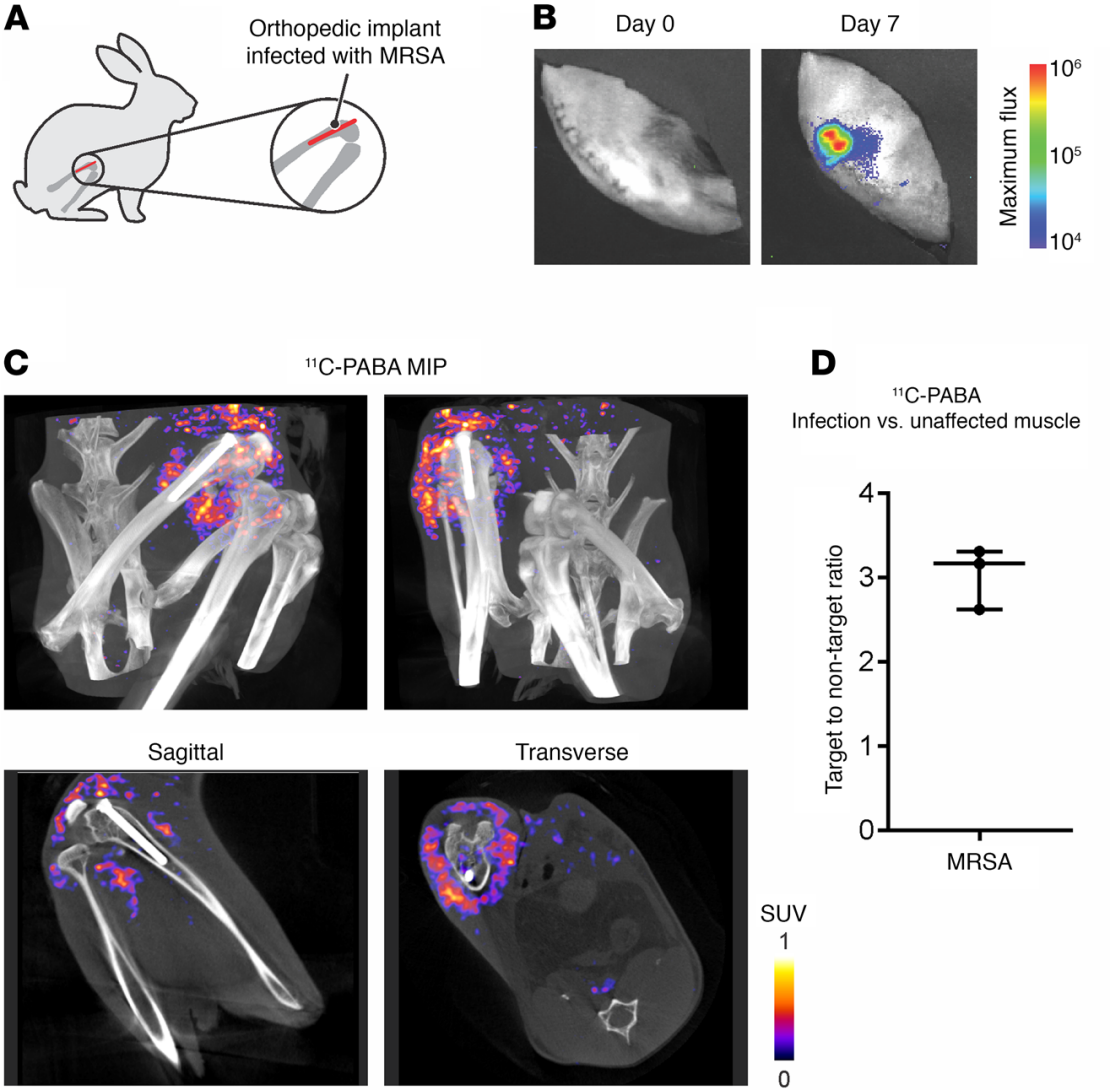
^11^C-PABA PET/CT imaging in a rabbit model of MRSA prosthetic joint infection. (**A**) A prosthetic metal implant was inserted into the femur of Dutch Belted rabbits and subsequently infected with MRSA (*n* = 3). (**B**) Progression of the infection was observed by optical imaging. Seven days after infection, the bioluminescent bacteria were visible over the knee. (**C**) Maximum intensity projection (MIP) and sagittal and transverse views of ^11^C-PABA PET/CT images from a representative rabbit where the ^11^C-PABA signal can be seen at the site of infection (purple to yellow). Minimum background is observed in the contralateral unaffected muscle and bone. (**D**) Quantification of the ^11^C-PABA PET signal, represented as median ± IQR of infection vs. unaffected muscle target-to-nontarget ratio.

To determine if ^11^C-PABA was able to selectively detect an infection associated with a prosthetic implant, a medial parapatellar arthrotomy was performed in Dutch Belted rabbits, in which an orthopedic-grade titanium implant was inserted (Figure 2A). During the procedure, the femoral canal was infected with bioluminescent MRSA. Optical imaging was performed at day 0 and day 7 after infection to confirm the presence of viable bacteria (Figure 2B). A contralateral sterile inflammation site was not generated in these rabbits to avoid limitations in mobility. Seven days after the surgical procedure, the ^11^C-PABA PET signal was observed in the infected bone, implant, and surrounding tissue (Figure 2C and Supplemental Figure 2). The TNT ratio for ^11^C-PABA PET at the infection site was 3.17 (IQR, 2.62–3.31) compared with the contralateral leg (Figure 2D). The bacterial burden after completion of imaging at the infection site was 7.74 ± 0.9 log_10_ CFU.

### Detection of vertebral discitis-osteomyelitis in a rat model.

**Figure 3:**
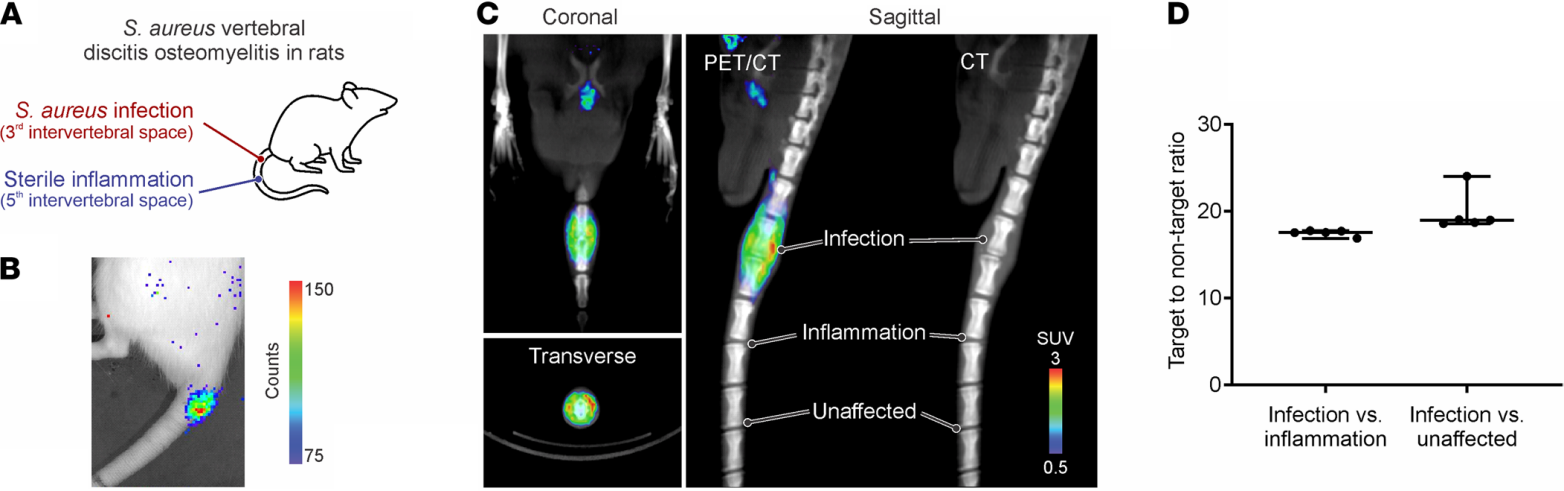
Imaging a vertebral discitis-osteomyelitis rat model using ^11^C-PABA. (**A**) In rats, the third intervertebral segments distal to the coat/tail transition were inoculated with live *S*. *aureus*. Heat-killed bacteria were injected into the fifth intervertebral space to induce sterile inflammation. (**B**) Optical imaging on day 3 after inoculation. Site of injection of bioluminescent *S*. *aureus* is visible and localized. (**C**) Coronal, transverse, and sagittal PET/CT images of ^11^C-PABA on day 4 after inoculation with bioluminescent *S*. *aureus*. Uptake of ^11^C-PABA is visible and localized at the site of live infection (third intervertebral space). The sagittal PET/CT image shows absent signal at site of sterile inflammation (fifth intervertebral space) or unaffected intervertebral disc space (seventh intervertebral space). (**D**) VOI analysis of ^11^C-PABA PET on day 4 after inoculation with bioluminescent *S*. *aureus* (*n* = 5). Data are represented as the median ± IQR of the infection vs. inflammation and infection vs. unaffected target-to-nontarget ratio.

^11^C-PABA PET was able to correctly detect the infection site in the bone of rats infected with *S*. *aureus* (Figure 3A). The bacterial burden after completion of imaging at the infection site was 7.6 ± 0.3 log_10_ CFU. Four days after infection, the uptake of ^11^C-PABA was visualized at the infection site (Figure 3, B and C) and the quantified TNT ratio of the infection compared with heat-killed control was 17.53 (IQR, 16.85–17.70) (Figure 3D). Minimal signal was observed in areas of sterile inflammation and unaffected tissues.

### Biodistribution and radiation dosimetry of ^11^C-PABA in healthy humans.

**Figure 4:**
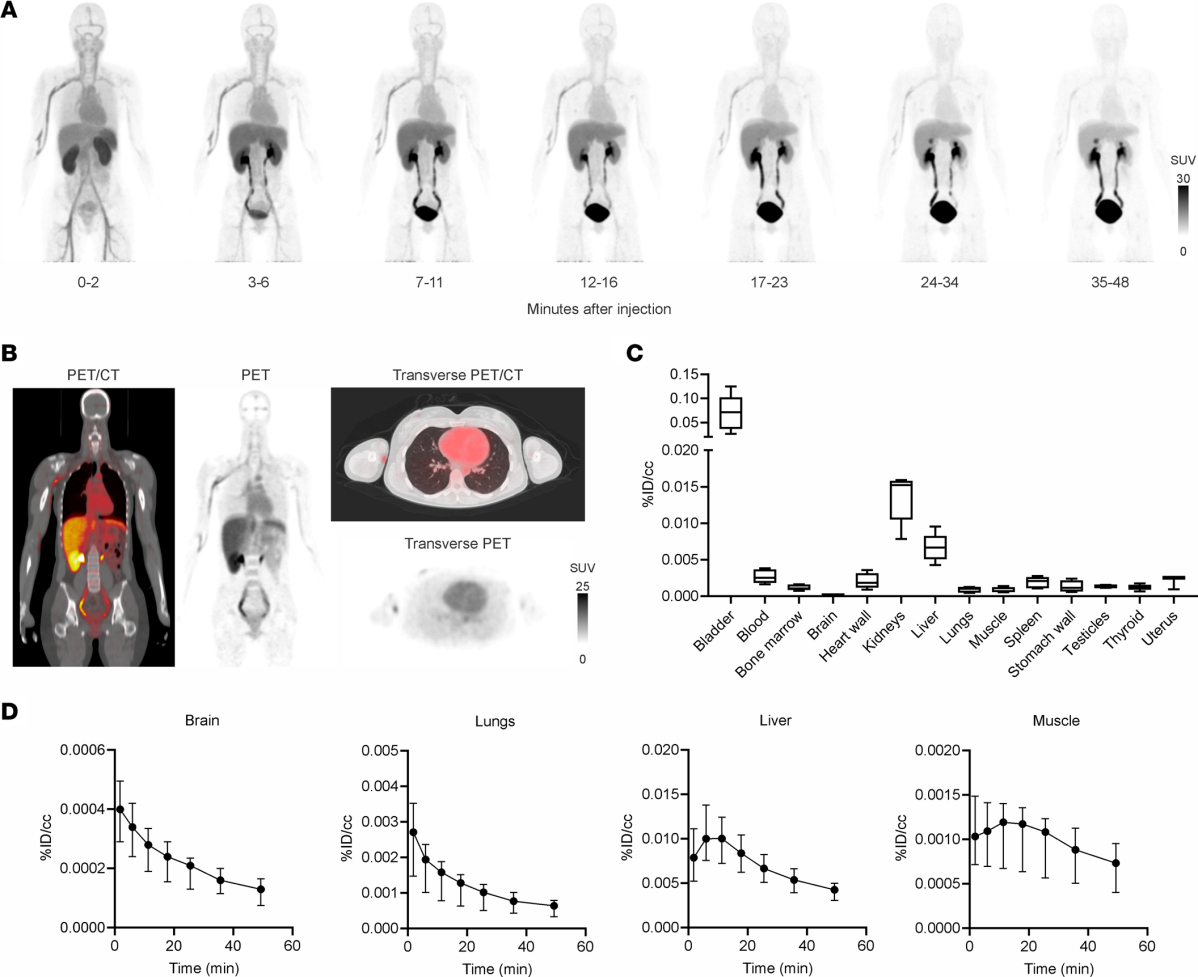
Biodistribution of ^11^C-PABA in healthy humans. (**A**) Sequential ^11^C-PABA PET maximum intensity projection of a representative healthy human participant. For this participant, ^11^C-PABA was injected into the median cubital vein of the left arm. All images were adjusted to the same mean SUV. (**B**) Coronal and transverse ^11^C-PABA PET/CT sections of participant no. 1 at 30 minutes after injection. (**C**) Tissue biodistribution of ^11^C-PABA 30 minutes after injection. Data are represented as the median percentage injected dose per cc (%ID/cc), IQR (boxes), and range (whiskers show minimum and maximum values) (*n* = 5 participants). (**D**) Time-activity curves of ^11^C-PABA in the brain, lungs, liver, and muscle of healthy humans. Data are represented as the median ± IQR (*n* = 5 participants).

Five healthy human volunteers (3 female and 2 male participants; age range, 22–34 years) were injected with 689.32 ± 15.36 MBq of ^11^C-PABA (Table 1). There were no adverse or clinically detectable pharmacologic effects in any of the 5 participants. No significant changes in vital signs were observed. After injection, ^11^C-PABA cleared rapidly from the circulation, predominantly via renal elimination (8) (Figure 4A and Supplemental Figure 3). Minimal signal was observed in the lungs, brain, and musculoskeletal tissues (Figure 4B). Quantification of the physical decay-corrected biodistribution of ^11^C-PABA in healthy participants showed increased signal in the bladder, followed by the kidneys and liver (Figure 4C). ^11^C-PABA had a rapid clearance from most organs providing an overall low background signal (Figure 4, C and D, and Supplemental Figure 4). The mean percentage of free radiotracer in plasma from all participants was 40.62% at 5 minutes after injection (Supplemental Table 1).

Time-integrated activity coefficients (TIACs) (i.e., residence times) were obtained by drawing VOIs corresponding to each organ identified in the longitudinal PET imaging data. The calculated TIACs for all participants are shown in Table 2. For both sexes, the longest TIACs were in the bladder (7.78 ± 2.15 min and 4.83 ± 0.09 min in women and men, respectively), liver (2.49 ± 0.21 min and 2.60 ± 0.04 min, respectively), kidneys (0.82 ± 0.18 min and 1.05 ± 0.08 min, respectively), and lungs (0.84 ± 0.33 min and 0.63 ± 0.07 min, respectively). Calculated TIACs for individual participants are shown in Supplemental Table 2. The urinary bladder received the highest absorbed dose and was the dose-limiting organ, with a mean absorbed dose of 0.10 ± 0.04 mSv/MBq (Table 3 and Supplemental Table 3). The effective dose was 0.01 ± 0.003 mSv/MBq. Given that the urinary bladder is the dose-limiting organ, modeling demonstrated that if the patient urinates 15 minutes after injection of ^11^C-PABA, the effective dose would be 1.8 times lower (0.004 ± 0.001 mSv/MBq).

### Metabolism of ^11^C-PABA.

PABA is predominantly conjugated to glycine to form *para*-aminohippuric acid (PAHA) by the glycine *N*-acyltransferase (9, 10). PABA also undergoes acetylation within the cellular cytosol via *N*-acetyltransferase I to form *para*-acetamidobenzoic acid (PAABA). Five minutes after intravenous injection of ^11^C-PABA in healthy human participants, only 2.18% ± 0.90% of the plasma radioactivity corresponded to ^11^C-PABA, with most of the compound being metabolized to ^11^C-PAABA (90.37% ± 5.20%), comparable to what had been previously reported with nonradiolabeled PABA (11) (Supplemental Figure 5, A and B). Similar results were noted in healthy rabbits, where 5 minutes after intravenous injection of ^11^C-PABA, 81.03% ± 2.30% of the radioactivity present in plasma was ^11^C-PAABA, with only 0.60% ± 0.26% of the plasma radioactivity corresponding to ^11^C-PABA (Supplemental Figure 5C).

Our results in rabbits suggest a rapid acetylation phenotype with PAABA as the main metabolite (12). Given the high affinity of *N*-acetyltransferase for PABA, we hypothesized that administration of 1 mg nonradiolabeled PABA just before the ^11^C-PABA injection would saturate the enzyme and slow down the rapid acetylation of ^11^C-PABA. Administration of unlabeled PABA 1 minute before the ^11^C-PABA injection increased the availability of ^11^C-PABA in plasma to more than 20% 5 minutes after injection in rabbits (Supplemental Figure 5C).

## Discussion

Bacteria are evolutionary and phylogenetically distinct from eukaryotic cells. This provides opportunities to leverage the fundamental biochemical differences between bacteria and mammalian cells for the discovery of novel molecules that could be developed into pathogen-specific agents. Over the last decade, multiple bacteria-specific PET imaging agents have been developed to target distinct metabolic pathways (13). These efforts have led to the characterization of radiolabeled sugars, such as ^18^F-FDS (7, 14), ᴅ-amino acids that get incorporated in the bacterial cell wall (15, 16), and other promising compounds (1, 2, 17).

Unlike multicellular eukaryotes, many unicellular organisms like bacteria synthesize folate rather than relying on dietary sources of this cofactor. Given how ubiquitous and conserved this pathway is among different bacteria, the incorporation of PABA into tetrahydrofolate by the bacterial dihydropteroate synthase has been considered as a promising target for pathogen-specific imaging. Inhibitors with nanomolar affinity to key enzymes within the folate synthesis pathway, such as radiolabeled trimethoprim (^11^C-TMP) and its analog fluoropropyl-trimethoprim (^18^F-FPTMP), have also been evaluated as bacteria-specific imaging agents (18, 19). PABA and its antibacterial structural analog, sulfonamide, enter bacterial cells by passive diffusion across the cell membrane (20). Previously, we have shown how radiolabeled PABA rapidly accumulates in a wide range of pathogenic bacteria and their respective clinical strains, including MDROs (3). We also performed pilot experiments to demonstrate that ^11^C-PABA was able to detect infection sites in a mouse model of *E*. *coli* myositis (4) and evaluated a radiofluorinated analog of PABA (2-^18^F-PABA) that could detect and localize *S*. *aureus* infections in a rat model and correctly monitored antibiotic response (5).

In this study, ^11^C-PABA was synthesized at high yield under cGMP conditions and was used to detect infections by *E*. *coli* and *S*. *aureus* in a rabbit myositis model. ^11^C-PABA PET was able to detect the site of disease with a PET signal 4–5 times higher in the infected site compared with sterile inflammation in the contralateral muscle. Similarly, in a *S*. *aureus* vertebral discitis-osteomyelitis rat model, ^11^C-PABA PET localized the infection site with minimal background signal. Given that PABA accumulates in a wide range of bacteria (3), ^11^C-PABA has a major advantage for clinical translation, as it can be useful to differentiate sites of infection over sterile inflammation.

^11^C-PABA PET was also able to correctly detect the site of disease in a prosthetic joint MRSA infection model. Since MRSA strains emerged in the 1960s, they have become a major clinical and epidemiological problem in hospitals and communities (21). In part owing to its capacity for biofilm formation, MRSA is one of the few pathogens routinely implicated in nearly every type of hospital-acquired infection (22). Treatment of a prosthetic joint bacterial infection, including MRSA, usually requires prolonged antibiotic therapy with multiple drugs and surgical debridement or resection arthroplasty with or without reimplantation of the prosthesis. Initiation of antibiotic therapy is usually delayed until the specimens for microbiological culture are obtained (via joint aspiration, joint debridement, and/or hardware removal). Therefore, having a noninvasive PET imaging agent such as ^11^C-PABA that can determine the presence of infection and its location within the prosthetic joint with high specificity would be a major advantage in the diagnosis and management of these patients.

^11^C-PABA PET imaging in healthy human participants was safe, well-tolerated, and without adverse effects in all volunteers. The biodistribution of ^11^C-PABA showed rapid renal elimination with overall low background activity in the remaining organs. Taking advantage of these optimal excretion kinetics, we recently evaluated ^11^C-PABA as a novel radiotracer for functional renal imaging (8, 23). The low background signal of ^11^C-PABA PET in the brain, lung, muscle, and heart could allow the optimal visualization of sites of infections within these organs. The dosimetry of ^11^C-PABA was also favorable, with low radiation exposures to most organs except the bladder. Radiation exposure could be reduced even further if the patient is asked to void after injection of ^11^C-PABA.

Our study has some limitations. First, PABA is mainly metabolized by acetylation and glycine conjugation in the liver, forming PAABA, PAHA, and PAAHA in humans, rabbits, and other species (12). Similar to that of other aromatic amine and hydrazine drugs, the acetylation phenotype of PABA is genetically determined, resulting in slow or fast acetylation (24). In our experiments with healthy rabbits, we observed a fast acetylation phenotype with rapid conversion of ^11^C-PABA into ^11^C-PAABA. However, even the low amounts of free ^11^C-PABA remaining after injection were enough to localize the site of infection and differentiate it from sterile inflammation. In healthy human volunteers, we also observed a fast acetylation pattern in all participants, with rapid conversion of ^11^C-PABA into ^11^C-PAABA. To increase the amount of free ^11^C-PABA that is available to bacteria after intravenous injection, Li et al. recently used ethyl 2-^18^F-4-nitrobenzoate (2-^18^F-ENB) as a prodrug that is converted in vivo into 2-^18^F-PABA (25). We used an alternative approach focused on enzyme kinetics to reduce the in vivo metabolism of ^11^C-PABA. Reduction of *N*-acetylation of PABA can be achieved by saturation or substrate inhibition of *N*-acetyltransferase I (26). In healthy rabbits, administration of unlabeled PABA before ^11^C-PABA increased the amount of ^11^C-PABA in plasma by more than 35-fold (>20%). These results need to be further validated in infected animals and patients. Of note, this would not affect bacterial uptake, due to the very high threshold needed to block uptake in bacteria (3). However, the metabolism of ^11^C-PABA and its heterogeneity would need to be considered for future human studies. Second, it should be noted that stress due to host-immunological responses or antibiotic treatments may alter bacterial metabolism. However, PABA, which is metabolized via the bacterial folate pathway, is not affected by the bacterial growth phase (3). Nonetheless, the bacterial burden in some chronic infections may be lower than the limit of detection of ^11^C-PABA PET.

In conclusion, ^11^C-PABA PET imaging is able to differentiate infection from sterile inflammation in advanced models of musculoskeletal infection. Preliminary studies in healthy human volunteers suggest that after an intravenous dose, ^11^C-PABA is safe, well-tolerated, and rapidly cleared from most organs with predominantly renal elimination. Therefore, ^11^C-PABA has the potential for clinical translation to detect and localize a broad range of bacteria. Additional clinical studies in patients with confirmed infections are needed to evaluate the role of ^11^C-PABA PET imaging in diagnosing and monitoring bacterial infections.

## Methods

Further information can be found in Supplemental Methods.

### Bacterial strains.

Bacterial strains for *E*. *coli* and *S*. *aureus* were obtained from ATCC. *E*. *coli* (ATCC 25922) and *S*. *aureus* (ATCC 25923), and the bioluminescent strains of *S*. *aureus* (SAP231 and Xen-29), were aerobically grown to absorbance at 600 nm of 1.0 in lysogeny broth (BD) at 37°C. Heat inactivation was performed for 30 minutes at 90°C (4, 14). Only live and metabolically active *S*. *aureus* SAP231 and Xen-29 emit light (27, 28). *S*. *aureus* Xen-29 was obtained from Perkin Elmer. The bioluminescent USA300 community-acquired MRSA strain SAP231 was previously derived from the clinical isolate NRS384 (29). *S*. *aureus* SAP231 possesses a stable bioluminescent construct integrated into the bacterial chromosome; therefore, it was not grown under selective antibiotic media (30). *S*. *aureus* SAP231 has been previously used and described in multiple murine and rabbit prosthetic joint infection models (30–33).

### Rabbit infection models.

A cohort of New Zealand white rabbits (female, bodyweight 2.5–3.0 kg) was obtained from Charles River and infected with 5 log_10_ CFU of *S*. *aureus* or *E*. *coli* in the right triceps. To induce sterile inflammation, a 10 times higher concentration of the respective heat-inactivated bacteria, compared with the level of live bacteria, was injected into the contralateral left triceps for each animal. To develop the prosthetic joint model, 4 Dutch Belted rabbits (males, 10–16 weeks old, bodyweight 2.04 ± 0.62 kg) were obtained from Robinson Services and individually housed. A medial parapatellar arthrotomy was performed followed by drilling into the femoral canal in the intercondylar notch. Subsequently, 4 log_10_ CFU of MRSA (bioluminescent strain SAP231) in 10 μL PBS were pipetted into the hollowed femoral canal and an orthopedic-grade titanium locking peg implant (2 × 24 mm, Zimmer Biomet) was inserted flush with the articular surface followed by closing of the surgical site (27). All the rabbits were euthanized after ^11^C-PABA PET/CT to quantify bacterial burden at the site of infection. The tissues were harvested, homogenized, and plated in Lysogeny Broth agar using serial dilutions. Adherent bacteria in the metallic implant were quantified by vortexing the implant for 2 minutes (7800*g*), followed by sonication for 10 minutes, vortexing again for 2 minutes, serial dilutions, and plating as previously described (27).

### Vertebral discitis-osteomyelitis rat model.

Sprague Dawley rats (male, 10–12 weeks old) were used for all experiments. The rats were injected with 50 μL bioluminescent *S*. *aureus* Xen-29 aerobically grown to an absorbance of 1.0 at 600 nm in Lysogeny Broth with 100 μg/L kanamycin. The rats were injected in the third intervertebral space from the base of the tail at 50% depth (based on the diameter of the tail). The infections were allowed to develop for 4 days. Quantification of the bacterial burden was performed by homogenization of the postmortem infected tail segment and agar plating of serial dilution of the rat tail homogenates. CFU were counted on the plates after 24 hours of incubation at 37°C.

### Optical imaging.

After infection, rats infected with *S*. *aureus* Xen-29 and rabbits infected with MRSA SAP231 were followed with optical imaging under isoflurane anesthesia. Bioluminescence imaging was performed using the IVIS Lumina III imaging system (Perkin Elmer) with a 5-minute exposure time as previously described (27, 32).

### Radiochemical synthesis of ^11^C-PABA.

^11^C-PABA was synthesized at the Johns Hopkins Hospital and UCSF PET radiotracer facilities as a sterile, pyrogen-free solution with high radiochemical purity and specific activity as previously described (34). For human studies, the ^11^C-PABA radiosynthesis at Johns Hopkins followed good-manufacturing practices.

### Preclinical PET/CT imaging.

After incubation of the infections (3 days for rabbit myositis, 7 days for rabbit joint infection), the rabbits were anesthetized and injected intravenously with 370 MBq ^11^C-PABA through the lateral ear vein. PET was acquired 30–60 minutes after tracer injection using the NanoScan PET/CT (Mediso) followed by a CT for attenuation correction and anatomical coregistration. For ^18^F-FDG and ^18^F-FDS PET, animals were injected intravenously with 18.5 MBq of the tracer and imaged 45 or 120 minutes after injection, respectively (14, 35). For rat experiments, on day 4 after infection, the rats were injected intravenously with 30 MBq ^11^C-PABA and imaged under isoflurane anesthesia on a Siemens Inveon microPET/CT system (Siemens) from 0 to 40 minutes with PET followed by CT. Image analysis was performed by drawing VOIs based on the CT with AMIDE 1.0.4. VivoQuant 2020 (Invicro) was used for data visualization.

### First-in-human study.

Five healthy volunteers (3 female and 2 male volunteers) between 22 and 34 years old, were recruited from the Johns Hopkins Hospitals between January and February 2019. Written informed consent was obtained from all patients, and deidentified PET/CT images are presented. The evaluation before the study included a medical history, a physical examination with vital signs, and laboratory tests (a comprehensive metabolic panel, including alkaline phosphatase, total bilirubin, liver transaminase levels, and renal function [blood urea nitrogen and creatinine]). Eligibility included a laboratory evaluation within 28 days before enrollment. Female participants were excluded if pregnant or lactating. Other exclusion criteria included participants with hypertension, diabetes mellitus, a body mass index lower than 18.5 kg/m^2^ or higher than 30 kg/m^2^, a family history of renal disease, or a urinary tract infection in the prior 6 months. Participants who had been treated with an investigational drug, an investigational biologic, or an investigational therapeutic device within 30 days before radiotracer administration were also excluded. Follow-up was performed by telephone 25 days after imaging to assess for any side effects or other issues.

### Clinical PET acquisition and image reconstruction.

Human participants underwent whole-body dynamic PET/CT after intravenous injection of 689.9 ± 15.4 MBq (range, 670.8–705.6 MBq) of ^11^C-PABA, corresponding to a mean and SD administered mass of 0.5 ± 0.10 μg (range, 0.41–0.66 μg). A mid-thigh to the vertex of skull multibed dynamic PET was acquired using a Biograph mCT 128-slice PET/CT device (Siemens Healthineers) operating in 3-dimensional emission mode. PET data were acquired immediately after tracer injection using a multibed dynamic protocol for a total of 50 minutes after tracer injection. A helical CT (120 kVp, 30 mAs reference, 0.5 s/rotation, 0.8 pitch) was acquired and used for attenuation correction of the PET data and anatomic coregistration to delineate organ boundaries. Patients were asked to void the urinary bladder immediately after completion of the scan. The images were analyzed with Mirada XD (Mirada Medical) and PMOD (PMOD Technologies) for dynamic quantification. VOIs were drawn manually using the CT images as a reference for dosimetry calculation. Time-activity curves were generated and used to calculate organ TIACs for each participant. The resulting TIACs provided input data for the calculation of organ absorbed doses and effective dose for each participant using OLINDA/EXM.

### Dosimetry calculations.

The MIRD Committee S-value methodology (36), as implemented in the OLINDA/EXM software package (37), was used to perform the absorbed dose calculations. The S-value methodology provides the absorbed dose to a target tissue as the sum of dose contributions from all the radioactivity-containing (source) tissues. TIACs (i.e. residence times) were obtained by drawing VOIs corresponding to each organ identified in the longitudinal PET imaging data. Absorbed dose calculations were performed for ^11^C using the calculated TIAC values for the source organ that each phantom requires as input. A detailed description of the dosimetry calculations is provided in the supplemental data.

### Plasma protein binding.

Plasma protein binding was determined by isolation of fresh plasma samples from participants by centrifugation (1500*g* for 10 min at 4°C) followed by loading of a 0.5 mL aliquot of plasma into an Amicon Ultra-0.5 30 kDa purification device (Sigma-Aldrich), which was centrifuged at 14,000*g* for 30 minutes at room temperature. The filtrate was then saved, and the remaining supernatant was recovered by centrifugation at 1000*g* for 2 minutes. The filtrate, recovered supernatant, and the filter itself were counted separately in a Wallac Compugamma automated γ-counter. The filtrate represented free or unbound radiotracer, whereas the supernatant represented protein-bound radiotracer (≥30 kDa protein bound). The filter device represented nonspecific binding, and this value was subtracted from both supernatant and filtrate values. Values are expressed as free radiotracer.

### Quantification of metabolites.

A separate group of healthy New Zealand white rabbits (female, bodyweight 2.5–3.0 kg) was injected with 185 MBq of ^11^C-PABA through the lateral ear vein. At 0, 5, 15, and 30 minutes after injection, blood was drawn from the contralateral central auricular artery. To determine the metabolism of ^11^C-PABA in healthy humans, blood samples were obtained from the arm contralateral to the injection site at 1, 2, or 5 minutes after injection of ^11^C-PABA, followed by additional samples at 10, 15, and 30 minutes after injection. Blood from both rabbits and humans was separated into plasma through high-speed centrifugation. Urine samples from human participants were obtained through voluntary voiding after the scan was completed. The relative percentage of parent radioligand in plasma and urine was determined by high-performance liquid chromatography coupled to a radiation detector (GABI*, Elysia-Raytest). Briefly, 0.2–0.5 mL plasma was loaded onto a 2 mL Rheodyne injector loop and directed to the capture column (packed with Phenomenex Strata-X 33 μm polymeric reversed-phase sorbent) and detectors with initial mobile phase (aqueous 0.2 M KH_2_PO_4_, pH = 3.3) at 2 mL/min. After 2 minutes of elution, an analytical mobile phase (13%–87% ACN-aqueous 0.2 M KH_2_PO_4_, pH = 3.3) was applied to direct ^11^C-PABA and its nonpolar metabolites to an analytical column (Phenomenex, 4.6 × 250 mm, Gemini Column, 10 μm) and detectors at 2 mL/min. The HPLC system was standardized using ^11^C-PABA and nonradioactive PABA, PAHA, and PAABA. HPLC chromatograms were integrated to provide the percentage of parent radiotracer relative to other peaks associated with its radiometabolite.

### Statistics.

Prism 9.1.2 (GraphPad) was used for the data analysis. PET data presented as median and IQR were compared using a 1-way ANOVA with Tukey’s multiple-comparisons test. *P* values of less than 0.05 were considered statistically significant.

### Study approval.

All animal protocols were approved by the Johns Hopkins University and the UCSF Biosafety, Radiation Safety, and Animal Care and Use Committees. Human studies were approved by the Johns Hopkins Institutional Review Board and performed under the supervision of the Radioactive Drug Research Committee program.

## Author contributions

AAO, DMW, and SKJ conceptualized and designed the study. AAO, MFLP, RJM, CARB, JML, and DAD performed preclinical experiments. DP analyzed the dosimetry data. WGL performed the metabolism analysis. DPH and RFD synthesized ^11^C-PABA. LSM provided logistical support for rabbit experiments. AAO, EWT, and SPR recruited and consented human participants. AAO, MFLP, DMW, and SKJ analyzed the data and performed statistical analysis. AAO and SKJ wrote the initial draft, and all coauthors edited the manuscript. DMW and SKJ provided funding and supervised the project.

## Supplementary Material

Supplemental data

## Figures and Tables

**Table 3:**
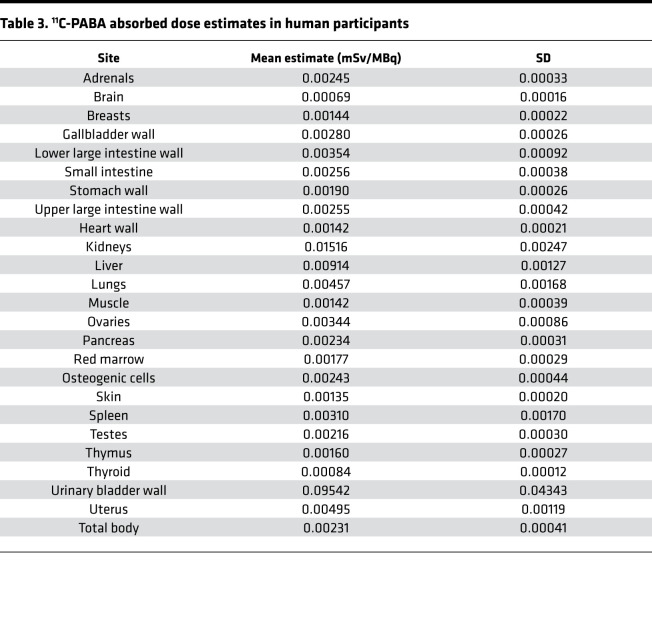
^11^C-PABA absorbed dose estimates in human participants

**Table 2:**
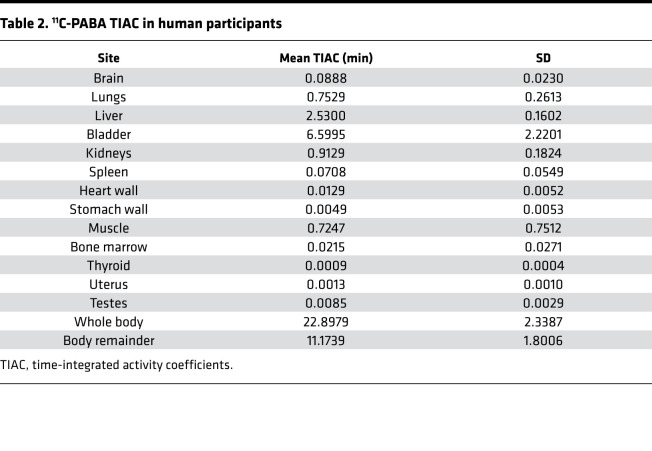
^11^C-PABA TIAC in human participants

**Table 1:**
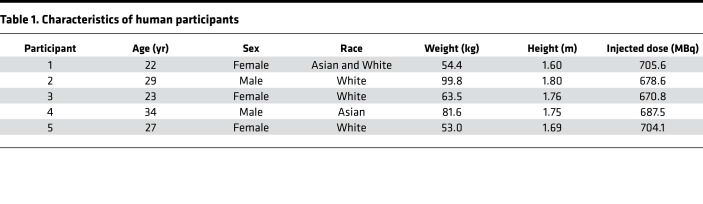
Characteristics of human participants

## Version 1 01/11/2022

Electronic publication
