# Supplementary material for: 11C-Para-aminobenzoic acid PET imaging of S. aureus and MRSA infection in preclinical models and humans
Source: JCI Insight. 2022 Jan 11;7(1):e154117. doi: 10.1172/jci.insight.154117 (PMC8765043; doi:10.1172/jci.insight.154117)
Supplement: Supplemental data [file jciinsight-7-154117-s183.pdf]

Supplemental Data

^{11}C-*para*-Aminobenzoic acid PET imaging of *S. aureus* and MRSA infection in preclinical models and human subjects

Alvaro A. Ordonez[1,2], Matthew F. L. Parker[3], Robert J. Miller[4], Donika Plyku[5], Camilo A. Ruiz-Bedoya[1,2], Elizabeth W. Tucker[1,6], Justin M. Luu[3], Dustin A. Dikeman[4], Wojciech G. Lesniak[5], Daniel P. Holt[5], Robert F. Dannals[5], Lloyd S. Miller[4], Steven P. Rowe[5], David M. Wilson[3], Sanjay K. Jain[1,2,5]

[1] Center for Infection and Inflammation Imaging Research, Johns Hopkins University School of Medicine, Baltimore, MD, USA

[2] Department of Pediatrics, Johns Hopkins University School of Medicine, Baltimore, MD, USA

[3] Department of Radiology and Biomedical Imaging, University of California, San Francisco, San Francisco, CA, USA

[4] Department of Dermatology, Johns Hopkins University School of Medicine, Baltimore, MD, USA

[5] Russell H. Morgan Department of Radiology and Radiological Sciences, Johns Hopkins University School of Medicine, Baltimore, MD, USA

[6] Department of Anesthesiology and Critical Care Medicine, Johns Hopkins University School of Medicine, Baltimore, MD, USA

Supplemental Methods

Dosimetry calculations

The MIRD Committee S-value methodology (1) as implemented in the OLINDA/EXM software package (2), was used to perform the absorbed dose calculations. The S-value methodology provides the absorbed dose to a target tissue as the sum of dose contributions from all the radioactivity-containing (source) tissues. The S-value is usually obtained by Monte-Carlo simulation that is based upon a standard or reference geometry of the patient and the decay characteristics and emission spectrum of the radionuclide. Once the absorbed dose for a series of organs is obtained, the effective dose may be calculated using tissue weighting factors.

Time-integrated activity coefficients (TIACs) (i.e. residence times) were obtained by drawing VOIs corresponding to each organ identified in the longitudinal PET imaging data. In most cases, the VOI covered the entire organ volume. For cases in which the entire organ volume could not be separated from adjacent structures, a partial VOI was drawn to estimate the organ concentration. Whole organ VOIs were drawn for the following organs of interest: brain, lungs, liver, urinary bladder, kidneys, spleen, heart wall, stomach wall, muscle, bone marrow, thyroid, uterus, and whole-body (outer contour of the whole field of view). A partial VOI was drawn for: heart wall (a partial VOI was drawn in the left ventricular wall only to estimate the activity concentration in the heart wall), red marrow (partial VOI drawn in the lumbar spine area), stomach wall, muscle, and uterus (because of the inability to distinguish the whole uterus on the PET image due to the high activity uptake in the bladder). The VOIs were drawn on the respective CT image providing a volume for each organ/VOI. For whole-organ VOIs the measured activity concentration was multiplied by the VOI volume to obtain total activity at the measured time-points. Time-activity concentration data for the drawn VOIs were recorded in PET units of Bq/ml for the measured time-points: (T0=2 min, T1=6 min, T2=12 min, T3=18 min, T4=26 min, T5=36 min, and T6=50 min). The activity concentration in each time-point was multiplied with the volume of the drawn VOI to calculate the total activity in Bq according to Equation 1:

$$\text{Activity concentration } \left(\frac{\text{Bq}}{\text{ml}}\right) \text{x mean VOI volume(ml)} = \text{Total Activity in VOI (Bq)} \qquad \textbf{Eq. 1}$$

The obtained activity-time curves were fit either by using a mono-exponential function, or a hybrid (trapezoidal + mono-exponential function to the last 4-5 data points) fit and then integrated to obtain the time-integrated activity. The resulting organ TIAs were divided by the injected activity to obtain TIAC or residence times for each source organ. The TIAC of the remainder of body was calculated by subtracting the sum of all the organs of interest TIAC from the whole body (whole field of view) TIAC. For the organs where a partial VOI was drawn the measured activity concentration was plotted as a function of time and integrated to obtain time-integrated activity concentration for the drawn VOI. Time-integrated activity for the whole organ was calculated according to Eq. 2:

$$\mathrm{TIA\ (Bq\ x\ seconds)} = \mathrm{TIA.conc.} \frac{\left(\frac{\mathrm{Bq}}{\mathrm{ml}} * \mathrm{s}\right)}{\mathrm{M_{VOI}}} / \mathrm{M_{VOI}}\ \mathrm{x}\ \mathrm{M_{tot}} \qquad \textbf{Eq. 2}$$

where $M_{voi} = d*V_{ref}$, $V_{ref,}$ and M_{tot} is the reference organ volume and mass derived from ICRP 60 (3) as implemented in OLINDA/EXM for female and male phantom organs as it applied to a specific patient in this study.

The absorbed dose to the urinary bladder (the dose-limiting organ in this study) was calculated using two methods: 1) the residence time for bladder was calculated from the measured time-points in patient images and used as explained above to obtain the absorbed dose to the bladder; 2) using the MIRD bladder model (4) to calculate the absorbed dose to the bladder for a 15 min voiding interval, the measured biological clearance half-time and a fraction of 1. The obtained TIACs for all source organs were used as input to OLINDA/EXM to obtain absorbed dose to the target organs. An adult female or adult male phantom was interchangeably used according to the sex of the patient. Absorbed dose calculations were performed for ^{11}C using the calculated TIAC values for the source organ that each phantom requires as input.

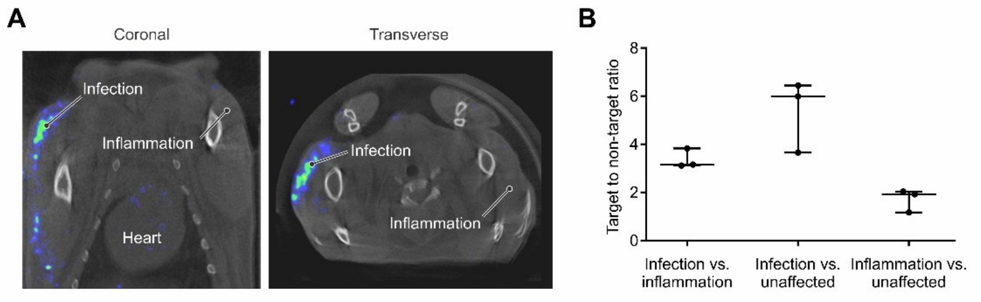

Supplemental Figure 1. ^{18}F-FDS PET/CT in the rabbit *E. coli* myositis model. The same cohort of *E. coli*-infected rabbits imaged with ^{11}C-PABA (Fig. 1), were injected with ^{18}F-FDS and imaged 120 min later (n=3). (A) The ^{18}F-FDS PET signal is observed in the site of infection with minimal background in other body sites, including the contralateral extremity with sterile inflammation. (B) ^{18}F-FDS PET had an infection vs inflammation TNT ratio of 3.16 (IQR, 3.14 to 3.49).

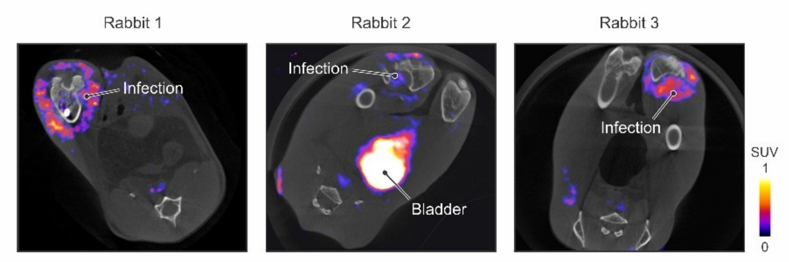

Supplemental Figure 2. ^{11}C-PABA PET/CT in a rabbit model of MRSA prosthetic joint infection. Three rabbits were imaged with ^{11}C-PABA PET/CT after surgical implantation of a prosthetic metal implant in the femur and subsequent infection with MRSA. Representative transverse PET/CT images are shown for each rabbit.

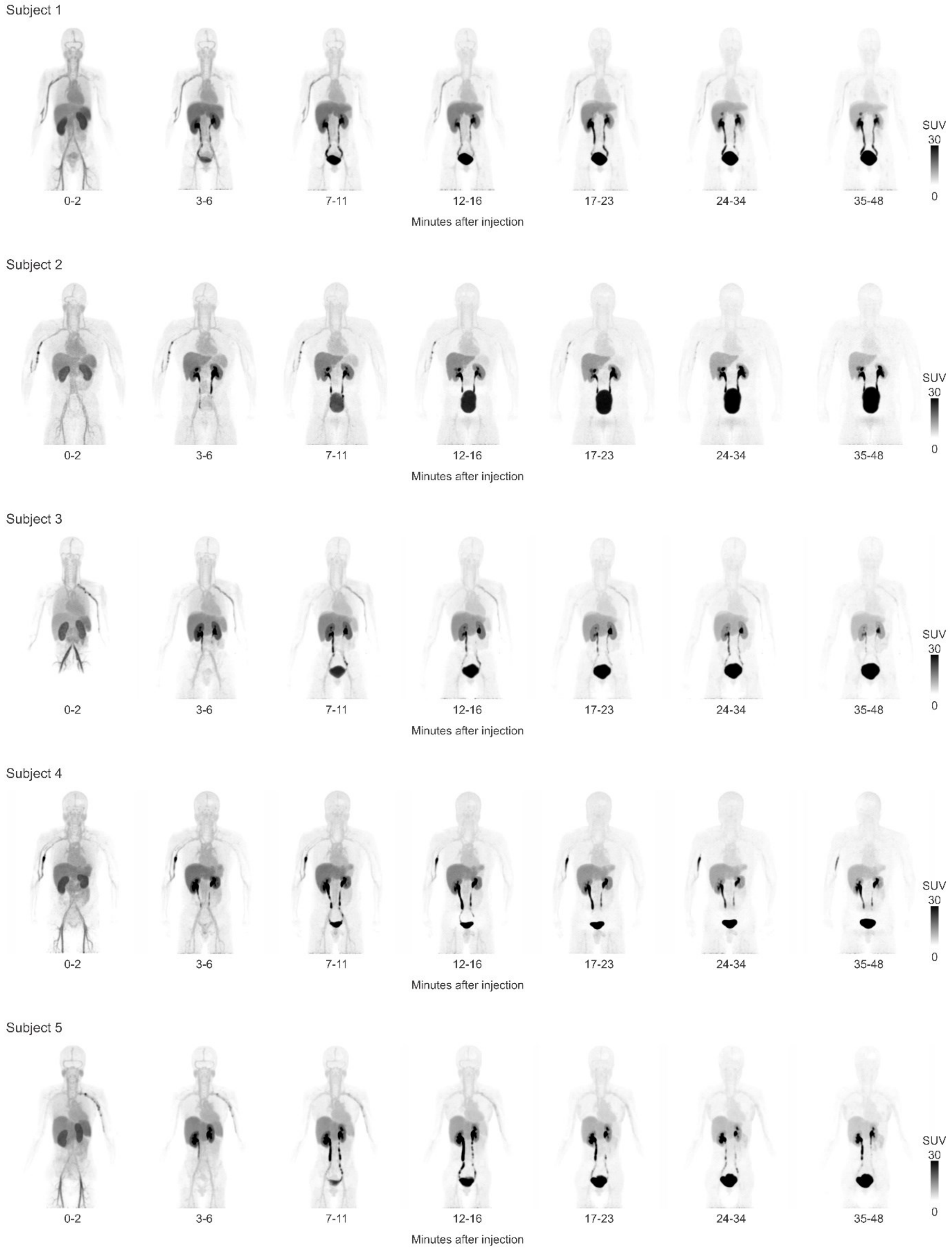

Supplemental Figure 3. ^{11}C-PABA PET in healthy human subjects. Sequential whole-body maximum-intensity-projection (MIP) images. All images were adjusted to the same mean SUV.

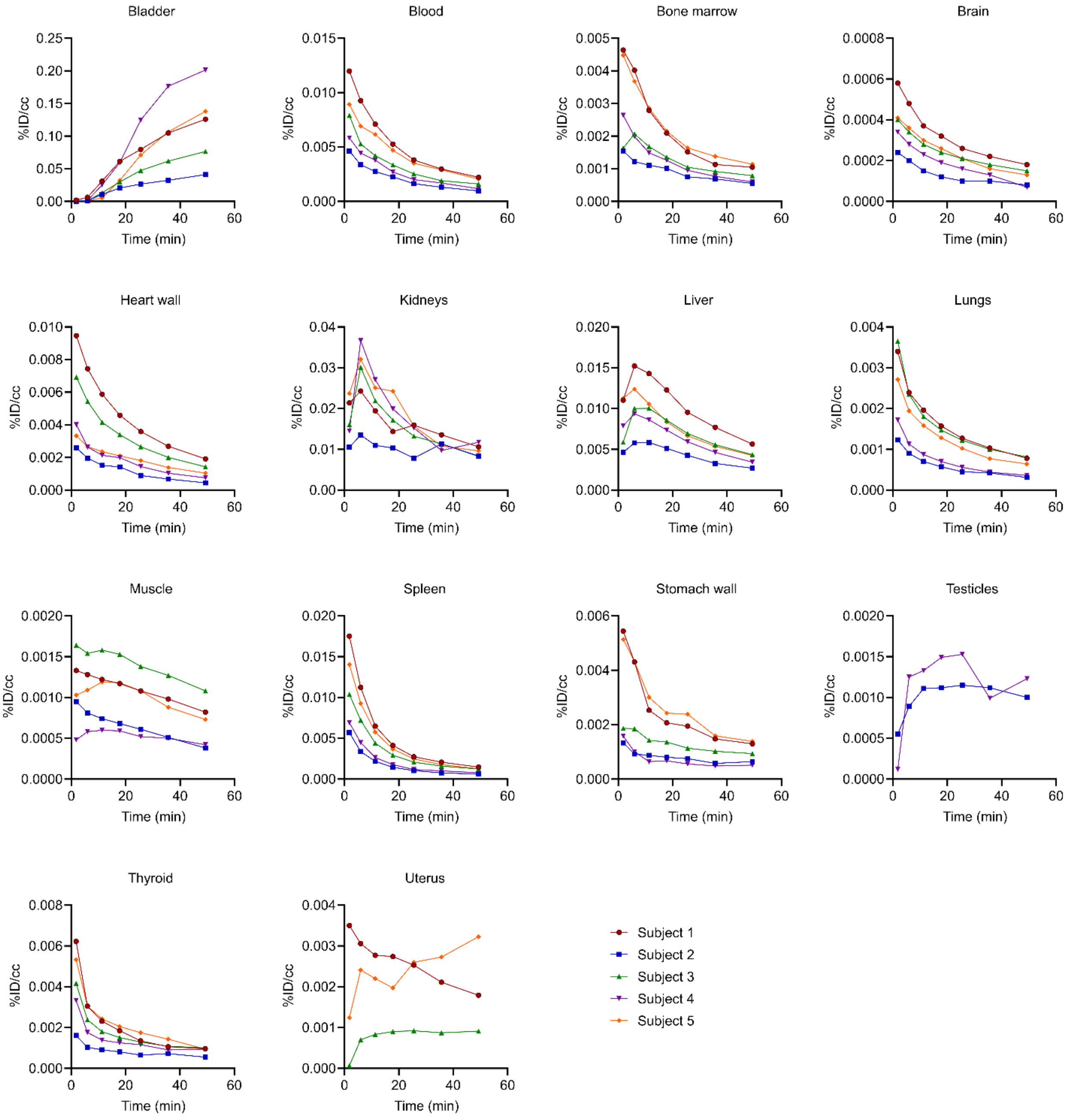

Supplemental Figure 4. Biodistribution of ^{11}C-PABA in healthy human subjects. A dynamic PET was performed after intravenous injection of ^{11}C-PABA. The mean organ uptake, represented as % injected dose per cubic centimeter (%ID/cc), is shown for all five subjects. Blood values were determined using a volume of interest in the left ventricle.

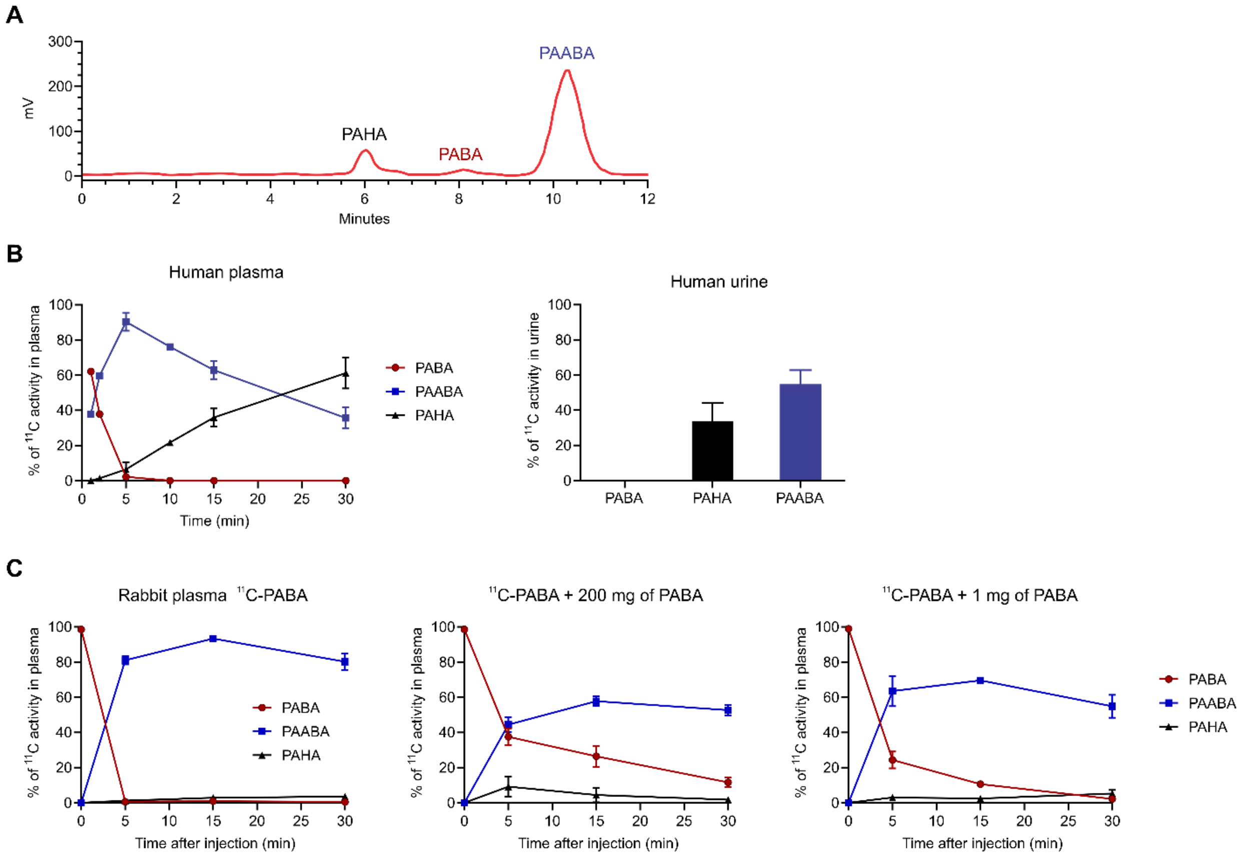

Supplemental Figure 5. Metabolism of ^{11}C-PABA in healthy humans and rabbits. (A) Representative radiochromatogram recorded for plasma collected 5 min after ^{11}C-PABA injection into a healthy human subject. (B) Quantification of ^{11}C-PABA and its metabolites in plasma and urine from healthy humans. (C) Quantification of ^{11}C-PABA and its metabolites in plasma of naive uninfected rabbits (left), rabbits injected with 200 mg of cold PABA prior to injection of ^{11}C-PABA (middle), and rabbits injected with 1 mg of cold PABA prior to injection of ^{11}C-PABA (right). Data represented as mean ± standard deviation. PAABA, *para*-acetamidobenzoic acid; PAHA, *para*-aminohippuric acid. Data from 5 human studies and a minimum of 3 rabbits per condition.

Supplemental Table 1. Plasma protein biding in human subjects. Plasma samples were collected 5 min post-injection of ^{11}C-PABA.

**Subject ID**	**^{11}C-PABA % protein binding** (mean ± SD)
1	54.7 ± 7.08
2	61.9 ± 1.27
3	60.2 ± 0.69
4	62.4 ± 4.66
5	57.7 ± 3.52

Supplemental Table 2. Calculated time-integrated activity coefficients (TIAC) or residence times for each source tissue from human subjects. Data represented in minutes. F1-F3, female subjects. M1-M2, male subjects. N/A, not applicable. SD, standard deviation.

	**TIAC (minutes)**								
	**Females**					**Males**			
**Tissue**	**F1**	**F2**	**F3**	**Mean**	**SD**	**M1**	**M2**	**Mean**	**SD**
Bladder	6.9203	10.2285	6.1898	7.7796	2.1521	4.7649	4.8938	4.8294	0.0912
Bone marrow	0.0685	0.0191	0.0126	0.0334	0.0306	0.0030	0.0040	0.0035	0.0007
Brain	0.1121	0.1009	0.0998	0.1043	0.0068	0.0549	0.0762	0.0655	0.0151
Heart wall	0.0059	0.0190	0.0105	0.0118	0.0067	0.0123	0.0168	0.0145	0.0032
Kidneys	1.0161	0.7901	0.6605	0.8222	0.1800	1.1064	0.9915	1.0490	0.0812
Liver	2.2966	2.7080	2.4536	2.4861	0.2076	2.5642	2.6277	2.5959	0.0450
Lungs	1.0641	0.9851	0.4584	0.8359	0.3293	0.6767	0.5803	0.6285	0.0682
Muscle	0.1828	1.8317	1.1838	1.0661	0.8307	0.2476	0.1773	0.2125	0.0497
Spleen	0.1368	0.0787	0.0307	0.0821	0.0531	0.1062	0.0015	0.0539	0.0740
Stomach wall	0.0016	0.0065	0.0135	0.0072	0.0060	0.0015	0.0014	0.0015	0.0001
Testes	N/A	N/A	N/A	N/A	N/A	0.0105	0.0065	0.0085	0.0029
Thyroid	0.0011	0.0005	0.0007	0.0008	0.0003	0.0010	0.0014	0.0012	0.0003
Uterus	0.0018	0.0002	0.0020	0.0013	0.0010	N/A	N/A	N/A	N/A
Whole body	25.1087	25.7379	20.7849	23.8772	2.6964	21.6980	21.1598	21.4289	0.3805
Remainder of body	13.3011	8.9695	9.6688	10.6465	2.3254	12.1487	11.7814	11.9651	0.2597

Supplemental Table 3. Absorbed dose estimates (mSv/MBq). Data represented as mean ± SD. F1-F3, female subjects. M1-M2, male subjects. N/A, not applicable. SD, standard deviation.

	Females					Males			
**Tissue**	**F1**	**F2**	**F3**	**Mean**	**SD**	**M1**	**M2**	**Mean**	**SD**
Adrenals	0.00299	0.00253	0.00234	0.00262	0.00033	0.00225	0.00215	0.00220	0.00007
Brain	0.00090	0.00074	0.00075	0.00080	0.00009	0.00050	0.00057	0.00053	0.00005
Breasts	0.00183	0.00139	0.00136	0.00153	0.00026	0.00134	0.00129	0.00132	0.00004
Gallbladder wall	0.00319	0.00294	0.00272	0.00295	0.00024	0.00260	0.00255	0.00258	0.00004
Heart wall	0.00159	0.00168	0.00135	0.00154	0.00017	0.00123	0.00124	0.00124	0.00001
Kidneys	0.01750	0.01390	0.01160	0.01433	0.00297	0.01730	0.01550	0.01640	0.00127
Liver	0.00941	0.01080	0.00974	0.00998	0.00073	0.00780	0.00793	0.00787	0.00009
Lower large intestine wall	0.00424	0.00467	0.00350	0.00414	0.00059	0.00266	0.00263	0.00265	0.00002
Lungs	0.00664	0.00614	0.00330	0.00536	0.00180	0.00360	0.00317	0.00339	0.00030
Muscle	0.00153	0.00198	0.00150	0.00167	0.00027	0.00106	0.00103	0.00105	0.00002
Osteogenic cells	0.00317	0.00242	0.00235	0.00265	0.00045	0.00214	0.00208	0.00211	0.00004
Ovaries	0.00409	0.00452	0.00340	0.00400	0.00057	N/A	N/A	N/A	N/A
Pancreas	0.00285	0.00239	0.00223	0.00249	0.00032	0.00218	0.00205	0.00212	0.00009
Red marrow	0.00221	0.00191	0.00167	0.00193	0.00027	0.00155	0.00150	0.00153	0.00004
Skin	0.00168	0.00138	0.00130	0.00145	0.00020	0.00121	0.00118	0.00120	0.00002
Small intestine	0.00301	0.00292	0.00243	0.00279	0.00031	0.00226	0.00220	0.00223	0.00004
Spleen	0.00540	0.00354	0.00196	0.00363	0.00172	0.00361	0.00099	0.00230	0.00186
Stomach wall	0.00231	0.00193	0.00190	0.00205	0.00023	0.00172	0.00163	0.00168	0.00006
Testes	N/A	N/A	N/A	N/A	N/A	0.00237	0.00195	0.00216	0.00030
Thymus	0.00206	0.00159	0.00154	0.00173	0.00029	0.00143	0.00138	0.00141	0.00004
Thyroid	0.00104	0.00075	0.00076	0.00085	0.00016	0.00078	0.00084	0.00081	0.00004
Upper large intestine wall	0.00311	0.00285	0.00249	0.00282	0.00031	0.00218	0.00213	0.00216	0.00004
Urinary bladder wall	0.10900	0.16000	0.09710	0.12203	0.03341	0.05480	0.05620	0.05550	0.00099
Uterus	0.00519	0.00691	0.00459	0.00556	0.00120	N/A	N/A	N/A	N/A

Supplemental References

1. Bolch WE, Eckerman KF, Sgouros G, and Thomas SR. MIRD pamphlet No. 21: a generalized schema for radiopharmaceutical dosimetry--standardization of nomenclature. *J Nucl Med.* 2009;50(3):477-84.
2. Stabin MG, Sparks RB, and Crowe E. OLINDA/EXM: the second-generation personal computer software for internal dose assessment in nuclear medicine. *J Nucl Med.* 2005;46(6):1023-7.
3. Recommendations I. ICRP Publication 60. *Annals of ICPR.* 1990;21(1-3).
4. Thomas SR, Stabin MG, Chin-Tu C, and Samaratunga RC. MIRD pamphlet no. 14 revised: a dynamic urinary bladder model for radiation dose calculations. *J Nucl Med.* 1999;40(4):102S.
